# TCDO: A Community-Based Ontology for Integrative Representation and Analysis of Traditional Chinese Drugs and Their Properties

**DOI:** 10.1155/2021/6637810

**Published:** 2021-09-23

**Authors:** Yan Zhu, Lihong Liu, Bo Gao, Jing Liu, Xingchao Qiao, Chaojie Lian, Yongqun He

**Affiliations:** ^1^Institute of Information on Traditional Chinese Medicine, China Academy of Chinese Medical Sciences, Beijing 100700, China; ^2^National Institutes for Food and Drug Control, Beijing 102627, China; ^3^University of Michigan Medical School, Ann Arbor, MI 48109, USA

## Abstract

Traditional Chinese drugs (TCDs) have been widely used in clinical practice in China and many other regions for thousands of years. Nowadays TCD's bioactive ingredients and mechanisms of action are being identified. However, the lack of standardized terminologies or ontologies for the description of TCDs has hindered the interoperability and deep analysis of TCD knowledge and data. By aligning with the Basic Formal Ontology (BFO), an ISO-approved top-level ontology, we constructed a community-driven TCD ontology (TCDO) with the aim of supporting standardized TCD representation and integrated analysis. TCDO provides logical and textual definitions of TCDs, TCD categories, and the properties of TCDs (i.e., nature, flavor, toxicity, and channel tropism). More than 400 popular TCD decoction pieces (TCD-DPs) and Chinese medicinal materials (CMMs) are systematically represented. The logical TCD representation in TCDO supports computer-assisted reasoning and queries using tools such as Description Logic (DL) and SPARQL queries. Our statistical analysis of the knowledge represented in TCDO revealed scientific insights about TCDs. A total of 36 TCDs with medium or high toxicity are most densely distributed, primarily in *Aconitum* genus, Lamiids clade, and Fabids clade. TCD toxicity is mostly associated with the hot nature and pungent or bitter flavors and has liver, kidney, and spleen channel tropism. The three pairs of TCD flavor-nature associations (i.e., bitter-cold, pungent-warm, and sweet-neutral) were identified. The significance of these findings is discussed. TCDO has also been used to support the development of a web-based traditional Chinese medicine semantic annotation system that provides comprehensive annotation for individual TCDs. As a novel formal TCD ontology, TCDO lays out a strong foundation for more advanced TCD studies in the future.

## 1. Introduction

Traditional Chinese medicine (TCM) is the traditional medicine that originated in China thousands of years ago. TCM is characterized by Chinese medical practices including traditional Chinese drugs, acupuncture, cupping therapy, gua sha, massage, bonesetter, qigong exercise, and dietary therapy. Traditional Chinese drugs (TCDs) not only include herbal or plant drugs but also include mineral and animal drugs. TCDs have been used to treat various diseases such as typhoid fever, chronic diseases, infectious diseases, and cancers [1]. TCDs have been used to treat COVID-19 and have also become a resource for drug discovery against COVID-19 [2]. TCDs have been widely used not only in China but also in many other regions and have been recognized as an attractive alternative to conventional medicine due to their valuable therapeutic efficacy [3–5].

TCDs are also sources or origins for developing many modern or Western types of chemical drugs. Many TCD-derived chemical drugs, such as artemisinin, digitoxin, quinine, and celastrol, are known to have remarkable effects in treating diseases. Artemisinin (qinghaosu), the first-line drug for malaria, was discovered by 2015 Nobel laureate Youyou Tu. Tu's discovery of artemisinin was inspired by the Chinese herb qinghao capable of combating the symptoms of malaria [6]. As another example, the discovery of ephedrine, an antiasthmatic and antibronchitis drug, was inspired by the clinical use of the Chinese herb ma huang to treat asthma for >4000 years [7]. Therefore, the deep understanding of TCDs would support modern chemical drug development.

There are different forms of TCDs. As a common form of TCDs, decoctions are usually preferred over harder herbal parts like roots, barks, and seeds. Decoction is a method of extraction by boiling herbal parts (e.g., herb, insect shell, and dried plants) to dissolve the chemicals of the materials. Decoction pieces (中药饮片) are processed materials to be ready for the decoction extraction. After a doctor prescribes the medicine, the assistant gives a patient decoction pieces. These pieces will then be used to make a soup, which will be drunk by the patient in order to cure the disease. Another form is Chinese patent drugs that are modernized and ready to use [8].

There are many databases and resources of TCDs. For example, the Traditional Chinese Medicine Integrated Database (TCMID; http://bidd.group/TCMID/), originally launched in 2013, is a comprehensive database of TCM/TCD modernization and standardization [9]. The Encyclopedia of Traditional Chinese Medicine (ETCM; http://www.tcmip.cn/ETCM/) includes many aspects of clinical and functional essential information on TCM herb species, formulas, and herbal ingredients [10]. SymMap (http://www.symmap.org/) is an integrative database of traditional Chinese medicine which is enhanced by symptom mapping [11]. While these databases provide useful TCD resources, no systematic and logical common data models or information representation formats are provided. A major bottleneck associated with these databases and resources is the disintegration and lack of interoperability of the data and knowledge of TCDs, their properties, and applications. Disintegrated and noninteroperable data and knowledge cannot be interpreted by computers, inhibiting computer-assisted semantic knowledge representation and reasoning.

Ontology is a structured vocabulary of human- and computer-understandable terms and relations that represent the entities and relations among the entities in a specific domain. Hundreds of biomedical ontologies [12, 13] have been developed and play increasingly important roles in standard data and knowledge representation, sharing, integration, and analysis. A major field of artificial intelligence (AI) is knowledge representation and reasoning (KR^2^, KR&R) and ontology is a foundation of KR&R and so AI [14]. Ontology provides a formal logic representation of the information in a specific domain. It also provides a logical framework for the organization and computer-interpretable study of the specific domain. A logical formal ontology is needed to support the integrative systematic data and knowledge representation, standardization, sharing, integration, and analysis of heterogeneous TCD knowledge and data. Therefore, an ontology for the TCD domain is expected.

Many ontology representations and ontology-based applications have been reported in the domain of TCM and TCDs. For example, Jang et al. presented a study in 2010 on their development of an ontology for medicinal materials based on traditional Korean medicine, which started with the expression of the relations among medical materials and patients' symptoms, diseases, and treatments [15]. In 2013, Basic Gu et al. reported their development of an ontology-oriented diagnostic system for traditional Chinese medicine based on relation refinement [16]. In their system, ontology was used to transform the implicit relations among syndromes into a machine-interpretable model and support diagnosis in traditional Chinese medicine. In order to explore the solution of integrating existing TCM terminologies, Long et al. reported an ontological framework in 2019 for the formalization and organization of the TCM knowledge [17]. Their system, called GFO-TCM, is based on the framework of the General Formal Ontology (GFO), a top-level ontology that integrates Object and Process. A formal middle-level ontology that is compatible with both the TCM terminology and modern medical terminology standards is then proposed [17]. Compared to GFO, the Basic Formal Ontology (BFO) [18] is another top-level ontology that has recently been accepted as an ISO/IEC top-level ontology standard 21838 (https://www.iso.org/standard/74572.html). BFO has been adopted by over 300 ontologies, with a major portion in the biological and biomedical fields, including in the community of Open Biological and Biomedical Ontology (OBO) Foundry [19]. In 2017, we reported a BFO-based ontology representation of traditional Chinese drugs against rheumatism [20]. A total of 26 traditional Chinese decoction piece drugs that have been traditionally used to treat rheumatism were represented. For each traditional Chinese drug, we collected its plant or animal source, the anatomical entity (e.g., root and stem), and the chemical entities identified from these drugs. The information was then ontologically represented and analyzed, leading to many insightful findings and hypotheses [20].

To better support TCD standardization and AI analysis, we have developed a community-based open-source TCD ontology (TCDO). TCDO is developed by aligning with the BFO, a well-recognized and widely used ISO top-level ontology. This report presents the initiation, development, and applications of the TCDO. We demonstrated our ontological structure that lays out a theoretical framework and practical usage for logically and systematically representing over 1,500 terms in TCDO. Many original proposals were laid out to support our high-level framework construction to study TCM and TCDs. Many new scientific insights are also presented as a usage of our TCDO system.

The rest of this paper will follow the standard paper format including Methods, Results, Discussion, and Conclusion. In the Methods section, we will introduce our TCD knowledge extraction and TCDO development, query, and applications. The Results section will cover the TCDO ontology scope, provenance, representative ontology term modeling, design pattern, statistics, and four TCDO-based use cases. TCDO related topics will then be discussed before the conclusion is provided.

## 2. Methods

### 2.1. Extraction of TCD Information

The TCD knowledge was obtained from many resources including Chinese Pharmacopoeia 2015 [21], Textbook of Chinese Materia Medica [22], and international standards from the World Health Organization (WHO) and International Standard Organization (ISO). These authoritative resources provide reliable and comprehensive TCD knowledge for our study. TCDs commonly used in clinical practice were manually selected and annotated by our domain experts in traditional Chinese medicine.

### 2.2. TCDO Development

TCDO is developed by following the OBO Foundry principles (e.g., openness and collaboration) [19] and using the eXtensible Ontology Development (XOD) strategy [23]. The XOD strategy includes four ontology development principles: ontology term reuse, ontology semantic alignment, ontology design pattern (ODP) usage for generating new terms and editing existing terms, and collaborative community effort. Specifically, TCDO development reuses and aligns with existing ontologies such as the NCBITaxon taxonomy ontology. OntoFox [24] was used to extract and reuse TCD-related terms from the existing ontologies. Ontorat [25] was used to generate new terms based on ontology design patterns (ODPs). Since we define TCDO as a community-based ontology, we have made TCDO an open-source ontology and invited the communities to participate in its further development and applications.

### 2.3. Translation between Chinese and English

The default language used to generate TCDO is English. Since TCDs are originally developed in China, we have also added Chinese to annotate the TCDs in TCDO. The method previously used for English-Chinese translation in the Cell Line Ontology (CLO) study [26] was also used for the differential representation. Specifically, the English term and Chinese term share the same ontology identifier but have their specific annotations. The language-specific term representation formats are as follows: *<rdfs:label xml:lang* *=* *“en” >English term</rdfs:label>* (for English term label) and *<rdfs:label xml:lang* *=* *“zh” >Chinese term </rdfs:label>* (for Chinese term label).

### 2.4. TCDO Ontology Home Page, Source, License, and Deposition

TCDO ontology source is freely available at GitHub: https://github.com/TCMOntology/TCDO.

The license of TCDO source is CC-BY (https://creativecommons.org/licenses/by-nc/4.0/). TCDO has been deposited to the BioPortal repository (http://bioportal.bioontology.org/ontologies/TCDO).

### 2.5. TCDO Query

Developed based on the DL and the Web Ontology Language (OWL) (https://www.w3.org/TR/owl-ref/), TCDO is computer-interpretable and can be queried using DL query (https://en.wikipedia.org/wiki/Description_logic). In this study, we performed the DL queries over the TCDO ontology under the platform of Protégé OWL editor [27]. HermiT Reasoner (http://www.hermit-reasoner.com/) was used before the DL query.

### 2.6. Toxicity Association Jaccard Index

The Jaccard index, also known as the Jaccard similarity coefficient [28], was used to calculate the similarities and associations between two data sets associated with TCDs (e.g., TCD nature and toxicity). For Jaccard index between properties *a* and *b* of TCDs, set A as the count of TCDs with property *a* and *B* as the count of TCDs with property *b*. Then, the measurement of Jaccard index is defined as the size of the intersection divided by the size of the union of the data sets as shown in the following formula:

In this study, we calculated the Jaccard Index between any of the TCD toxicities (including low, medium, and high) and specific TCD properties (including nature, flavor, and channel tropism). The data set for this analysis includes the toxicities and properties of all the TCDs covered in this study.

## 3. Results

### 3.1. Scope, Coverage, and Provenance of TCDO

TCDO is a community-based biomedical ontology in the domain of TCDs. TCDO aims at providing an open and public reference ontology for many application scenarios such as TCD terminology standardization, network pharmacology analysis, and clinical data standardization and analysis. Instead of covering all areas of the TCM, TCDO focuses on the ontological representation of all possible TCDs, the properties of each TCD, and the semantic relations among TCDs. Our focus on TCDs instead of all topics in TCM made TCDO more manageable and feasible to develop in a finite time range. In general, the topics related to TCDs include TCD decoction pieces (TCD-DPs), Chinese medicinal materials (CMMs), and Chinese patent drugs. Our current TCDO focuses on the ontological representation of TCD-DPs and CMMs. Chinese patent drugs are another major category of TCDs that we plan to cover in TCDO in the future.

The current TCDO development has two points of focus. First, we have systematically surveyed and defined high-level terms related to TCD, semantic relations, and the design patterns that interlink different terms. By comparing different top-level ontologies, we have chosen the BFO as our top-level ontology and aligned our TCD-related terms and definitions to the BFO. The alignment with the BFO allows us simultaneously to integrate TCDO with the other hundreds of ontologies. Second, we have applied the basic TCDO design to specifically represent over 500 TCD-DPs and over 400 CMMs, which represent more than 90% of TCDs that are introduced in Chinese Pharmacopoeia 2015 [21], Textbook of Chinese Materia Medica [22], and international standards from the WHO and ISO. TCDO has systematically represented TCD-DPs and CMMs. The properties of each TCD-DP and CMMs, including their nature, flavor, toxicity, and channel tropism, have been manually selected and represented in TCDO.

The knowledge represented in the TCDO was manually selected, annotated, and reviewed by our domain experts in traditional Chinese medicine. The correctness of the TCDO contents is supported by the usage of authoritative TCD resources and active participation by our experts with complementary backgrounds as described above. Only those contents supported with our domain experts' consensus are included in TCDO. In addition to natural language definitions, TCDO also provides semantic axioms that interlink different entities (such as hierarchical TCDs and their specific properties) in a logical way. In addition, we frequently accept feedbacks from our ontology users and make updates to feed different applications.

The TCDO ontology development uses a community-based ontology development strategy. The term “community-based ontology” means that the ontology and TCD communities have an active role and participate in adding terms, giving comments, and addressing issues that matter to them. Being a community-based ontology, TCDO also reuses many terms from existing community-based reference ontologies. We have also made the TCDO an open source and allowed users to access the source and submit issues to our GitHub website for community-level discussion. This approach still requires the manual construction and review from domain experts. In addition, our community-based approach invites communities to actively design and develop the ontology.

### 3.2. Key TCDO Term Modeling and Definitions

**Figure 1:**
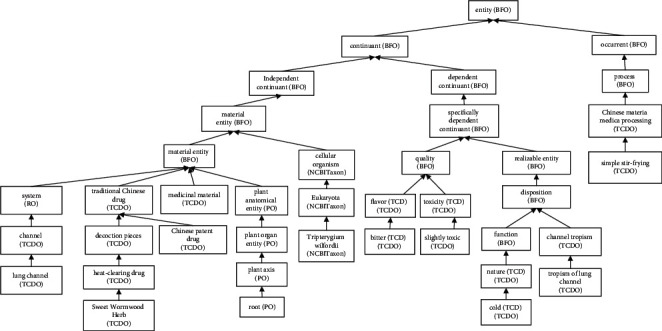
TCDO top-level hierarchy.

TCDO uses the BFO [18] as the top-level ontology and aligns all other terms in TCDO with BFO. Basically, BFO contains two branches: “continuant” and “occurrent” (Figure 1). The term “continuant”' represents time-independent entities such as material entity and data. The term “occurrent” represents time-related entities such as processes and time. We have carefully studied BFO and confirmed that the ontological representation of BFO fits very well with the TCDO scope and rationale. BFO has been used by more than 300 ontologies as the top-level ontology. The usage of BFO allows the interoperability and integration of TCDO with these other BFO-aligned ontologies seamlessly.

A major task of TCDO is to define the key TCDO terms that are associated with TCDs. Here we present our formal TCDO definition of several key TCD-related terms (“def.” is the abbreviation of “definition”):

“Traditional Chinese drug (TCD)” (“传统中药” in Chinese, TCDO_0000001) = def. is a drug that is developed originally from ancient China. A TCD is a drug derived from the usage of medicinal material grown and produced from natural world.

“Decoction piece” (“中药饮片” in Chinese, TCDO_0000002) = def. is a traditional Chinese drug that is prepared with crude medicine as raw materials. According to Chinese medicine theory, after processing, it can be directly used in traditional Chinese medicine clinical or pharmaceutical production and use of prescription drugs. It is also called medicinal slices or prepared drug in pieces.

“Chinese patent drug” (“中成药” in Chinese, TCDO_1000000) = def. is a traditional Chinese drug that is modernized into a ready-to-use form, such as tablets, oral solutions, or dry suspensions.

“Medicinal material” (“中药药材” in Chinese, TCDO_1000068) = def. is a material entity that is rude natural medicinal for processing and preparing traditional Chinese drugs. Medicinal materials are medicinal parts of medicinal plants, animals, and minerals after preliminary processing, which are used as raw materials to make decoction pieces in Chinese medicines. They are also called Chinese crude drug or Chinese Materia Medica (CMM) (ISO18662—1: 2017).

TCD has many special features or properties. We used the BFO framework to represent the four unique TCD features: “TCD nature” (“中药药性” in Chinese, TCDO_0000063), “TCD flavor” (“中药药味” in Chinese, TCDO_0000064), “channel tropism” (“归经” in Chinese, TCDO_0000062), and “TCD toxicity” (“中药毒性” in Chinese, TCDO_0000065). The TCD flavor and toxicity are defined as BFO: quality, TCD nature is defined as BFO: function, and channel tropism is defined as BFO: disposition.

First, the TCD medicinal nature is considered as the function of drugs. In TCM, hot, warm, cold, and cool are the four natures of TCDs. We define these four natures as the subclasses of BFO: function because they reflect the action tendency of TCDs in the body to regulate the physiological “cold” and “heat” changes and balance in the body. For example, the TCDs with the hot nature tend to inhibit the disease with cold nature. Cold and cool drugs have the actions of relieving or removing heat syndrome and can generally be used for clearing away heat, eliminating pathogenic fire, and detoxicating. Warm and hot drugs relieve or remove cold syndrome and can generally be used for dispelling cold, warming the interior, and invigorating yang [29]. Specifically, the TCD nature is defined as follows:

“TCD nature” (“中药药性” in Chinese, TCDO_0000063) = def. is a function of TCD which induces cold and heat changes in the body according to the cold or heat property of the diseases treated based on traditional Chinese medicine. There are four TCD natures, cold, hot, warm, and cool, which are summarized mainly from the body's response to traditional Chinese drugs [30].

Second, the Phenotype and Trait Ontology (PATO; https://github.com/pato-ontology/pato/) defines flavor as “a quality of a physical entity inhering in a bearer by virtue of whether the bearer's molecules are being perceived by a taste and odorant receptors.” Our TCD flavor (including astringent, bitter, pungent, salty, sour, sweet, and tasteless) is considered as subclass of the PATO: flavor (http://purl.obolibrary.org/obo/PATO_0000043). Specifically, “TCD flavor” is defined in TCDO as follows:

“TCD flavor” (“中药药味” in Chinese, TCDO_0000064) = def. is a flavor that reflects the common function of decoction pieces in a highly concentrated and abstracted way.

Third, the Relation Ontology (RO) defines a “system” (RO_0002577) as “a material entity consisting of multiple components that are causally integrated.” In TCM, channel (经絡) is a system of conduits which connects different parts of body (e.g., the bowels, viscera, extremities, superficial organs, and tissues) through qi and blood, making the whole body an organic whole. Tropism is the turning of whole or parts of an organism in a particular direction in response to an external stimulus. Channel tropism refers to the tendency of having selective therapeutic effects of a drug on some parts of a human body in preference. A drug may elicit evident or specific therapeutic action on the pathological changes in one or several channels [31]. Correspondingly, channel tropism is defined as follows:

“Channel tropism” (“归经” in Chinese, TCDO_0000062) = def. is a disposition that the TCD tends to have therapeutic effects on the pathological changes in one or several certain channels. It is also called meridian entry.

Lastly, we define TCD toxicity as a subclass of OAE: “drug toxicity” (OAE_0001804). In the Ontology of Adverse Events (OAE) [32], drug toxicity is defined as a quality that represents the level of critical or lethal reaction to a dosage of a drug medication. Accordingly, the TCD toxicity is defined as follows:

“TCD toxicity” (“中药毒性” in Chinese, TCDO_0000065) = def. is a quality that represents the level of critical or lethal reaction to a dosage of a TCD drug medication.

The Supplemental PDF File (Supplemental Table 1) lists the name, name in Chinese, ID, and text definitions of these terms above in tabular form.

### 3.3. TCDO Design Patterns and Demonstration of the Representation of Chinese Medicinal Material and TCD Decoction Pieces

**Figure 2:**
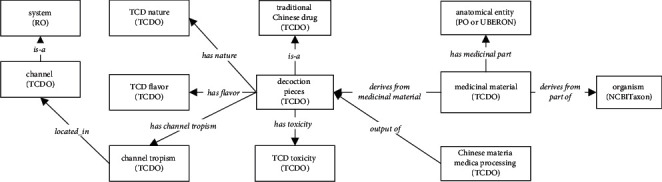
General TCDO ontology design pattern. Decoction pieces and their properties are modeled.

Figure 2 shows the high-level TCDO design pattern. Specifically, decoction pierces are considered as a TCD and derive from medicinal material, which is further derived from an anatomical entity and an organism. Each drug of decoction pieces has four types of properties: toxicity, nature, flavor, and channel tropism. Each channel tropism is defined to be located in some channel which is part of the system in traditional Chinese medicine.

To illustrate how TCDO systematically and logically represents a TCD, we use the honey ephedra (ma huang) and ephedrae herba [33] as examples. Ephedra is a Chinese shrub which has been used for medicinal purposes in China for thousands of years.

**Figure 3:**
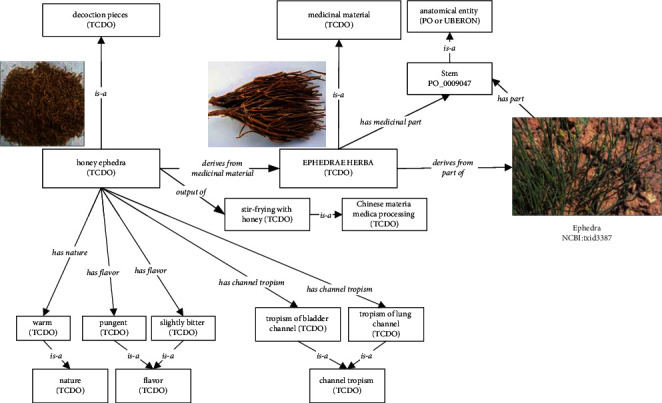
Diagrammatic representation of honey ephedra (ma huang) and ephedrae herba in TCDO.

Figure 3 shows the design of how TCDO presents honey ephedra (ma huang) and ephedrae herba, which aligns with the general design pattern as shown in Figure 2. Specifically, the ephedrae herba is a decoction pieces TCD. The honey ephedra (ma huang) pieces TCD is derived from ephedrae herba as the medicinal material. Furthermore, ephedrae herba derives from the stem of some *Ephedra* (NCBITaxon_3387). Here ephedra is a term imported from the NCBI taxonomy ontology. The ma huang decoction pieces TCD is the specified output of the stir-frying with honey, a Chinese material medical processing. In terms of the properties of the honey ephedra (ma huang) pieces TCD, it has warm nature, pungent and slightly bitter flavor, and the bladder and lung channel tropisms (Figure 3).

**Figure 4:**
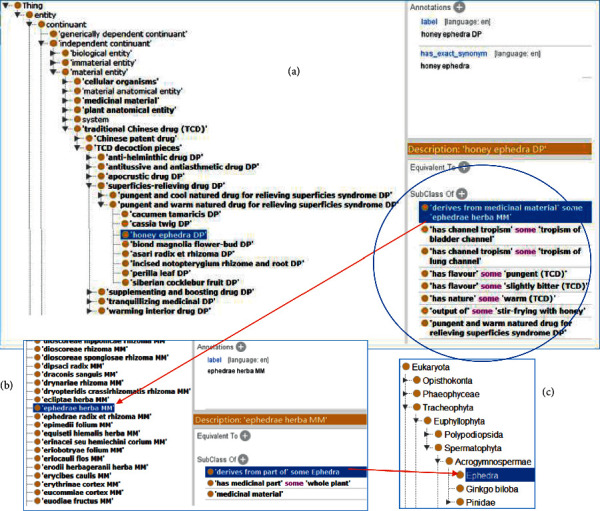
Ontological representation of honey ephedra (ma huang) and ephedrae herba in TCDO.

Figure 4 shows the screenshots of how the design related to honey ephedra (ma huang) and ephedrae herba in Figure 3 is laid out in TCDO. At the high level, the decoction pieces TCDs are classified based on their roles such as antihelminthic and tranquilizing medical roles. Honey ephedra DP TCD is defined to have the superficies-relieving drug role. The usage of the NCBITaxon taxonomy ontology for the representation of the source of Chinese medicinal materials establishes a link between traditional medicine and modern biomedical science.

### 3.4. The Statistics of TCDO Terms and Contents

We constructed the TCDO ontology manually by domain experts to support standardized TCD representation and integrated analysis. The properties of TCDs including nature, flavor, toxicity, and channel tropism (meridian entries) are also defined formally by logical relations.

As of July 28, 2020, TCDO contained over 1,500 terms with unique identifiers, including terms drawn from existing ontologies and more than 1,000 TCD-specific terms, labeled in both Chinese and English. We demonstrate its utility by its application for mining and investigating the correlation between plant species and properties of TCDs, leading to the discovery of important scientific insights and potential clinical applications.

TCDO has systematically represented 507 popular TCD-DPs and 435 CMMs, which were manually selected and annotated from authoritative textbooks, standards, and terminologies as detailed in the Methods section. The TCD-DP entities are categorized in 23 upper level terms by their clinical effects.

### 3.5. Four Use Cases

Below we demonstrate how TCDO can be used for scientific analysis and query.

#### 3.5.1. Use Case 1: TCDO-Based Query

**Figure 5:**
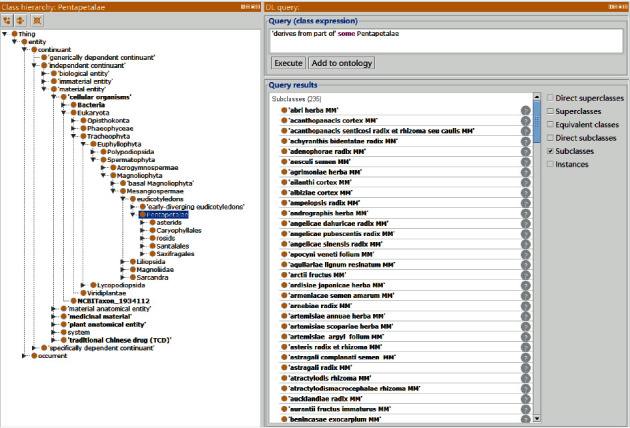
A DL query for identifying those medicinal materials developed from part of Pentapetalae. A total of 234 medicinal materials were identified from TCDO.

As a machine-understandable format, TCDO can be processed through different computational programs, such as the DL query and SPARQL.  Figure 5 shows a DL query use case that identified those medicinal materials developed from part of Pentapetalae, a taxonomic clade of flowering plants that have five petals. Note that, in nature, Pentapetalae are an unranked flowering plant subset of Gunneridae (core eudicots). This query identified 234 medicinal materials that are derived from part of Pentapetalae.

Another example is that we can use DL query to identify the number of TCDs that have both warm nature and toxicity:

      (“has nature” some “warm (TCD)”) and (“has toxicity” some “TCD toxicity”)
  Its equivalent SPAQL query is as follows:
    PREFIX owl: <http://www.w3.org/2002/07/owl#>
    PREFIX tcdo: <http://OntoTCM.org.cn/ontologies/>
      SELECT distinct ?subject
      WHERE {
       ?subject rdfs: subClassOf ?restriction1.
         ?restriction1 owl:onProperty tcdo: TCDO_0000321. # “has nature”
         ?subNature rdfs: subClassOf tcdo: TCDO_0200003. #“warm (TCD)”
          {?restriction1 ?restrictionPredicate1 ?subNature} UNION {?restriction1 ?restrictionPredicate1 tcdo: TCDO_0200003}.
       ?subject rdfs: subClassOf ?restriction2.
         ?restriction2 owl: onProperty tcdo: TCDO_0000323. #“has toxicity”
         ?subToxicity rdfs: subClassOf tcdo: TCDO_0000065. #“TCD toxicity”
       ?restriction2 ?restrictionPredicate2 ?subToxicity.
      }

Using such queries, we can quickly identify 22 decoction pieces TCDs that have both the nature of warm and any type of the three toxicity types (i.e., low, medium, and high toxicity) (Table 1). Such a method was used to automatically generate the results in Table 1–3.

#### 3.5.2. Use Case 2: TCDO-Based Analysis of TCD Toxicity and Its Relation with Taxonomy, Nature, Flavor, and Channel Tropism

Overall traditional Chinese drugs, including the large amounts of herbal products, are safe. However, a small portion of TCDs may not be. Based on the information of CMM's species mapped to NCBITaxon, the species of TCDs with different toxicities and their hierarchical ancestors were counted and compared. Our results showed that severely toxic TCDs focus on *Aconitum* genus, Asterids clade and Rosids clade, and Pentapetalae clade. These taxonomy branches cover most toxic TCDs. Considering that TCDs with toxicity are usually effective for some chronic diseases (e.g., cancer and rheumatism), the statistic results can be useful for predicting undiscovered toxicity of TCDs and finding new effective TCDs.

Table 2 shows the relation between TCD toxicity and the organism taxonomy. We found that 36 TCDs with medium or high toxicity are most densely distributed, primarily in *Aconitum* genus, Lamiid*s* clade, and Fabids clade. The plants with medium toxicity are most loosely distributed in different taxonomic groups, primarily in Mesangiospermae and some in Acrogymnospermae. In Mesangiospermae, the major category with medium or low toxicity exists in *Pentapetalae* under Eudicotyledons. Pentapetalae indeed is the taxonomical group with the highest number of medicinal plants. Fabids include medicinal plants with low, medium, or high toxicity. Fabids include the highest number of toxic medicinal plants, and Asterids have the second highest number of toxic medical plants.

Table 1 shows the Jaccard Index between TCD toxicity and the three TCD properties (nature, flavor, and channel tropism). In terms of TCD nature, it appears that the hot nature of TCDs is most likely related to high toxicity, followed by warm and cold. The TCDs with warm or cold natures have mostly medium toxicity. For TCD flavor, the TCDs with high and medium toxicity are mostly associated with pungent or bitter flavors. Regarding TCD channel tropism, TCDs with high toxicity mostly have liver, kidney, and spleen channel tropism. The TCDs with medium toxicity are mostly with liver, lung, spleen, large intestine, heart, and stomach channel tropisms. The low-toxicity TCDs mostly have liver channel tropism.

Table 3 lists 15 TCDs with the hot nature and their associated properties. Among the 15 hot TCDs, 5 have high toxicity, 2 medium toxicity, 1 low toxicity, and 7 no toxicity. Overall, the association between the hot nature and toxicity (8 out of 15) is high. All the toxic and hot TCDS also have the pungent flavor and may meanwhile have other flavors such as bitter flavor. The hot TCDs with liver, kidney, and spleen channels are mostly associated with the toxicity.

#### 3.5.3. Use Case 3: TCDO-Based Analysis of the Correlations between Flavor and Nature

We further examined the correlation by calculation of Jaccard Index between different flavors and natures of our collected TCDs (Table 4). Our results show that, out of 168 hot TCDs, 95 are pungent. So the pungent flavor is closely associated with warm nature. Sweet flavor is closely associated with neutral. Bitter flavor is closely associated with cold. These also verified the names of “bitter-cold medicine” and “pungent-warm medicine.” Interestingly, those cool or hot TCDs are not classified as sour. Overall, the three pairs of flavor-nature associations (i.e., bitter-cold, pungent-warm, and sweet-neutral) were identified from our systematical analysis of the knowledge stored in the TCDO.

#### 3.5.4. Use Case 4: TCDO-Based Annotation System

We have now applied TCDO to support the development of TCM semantic annotation system (TCM-SAS) that provides comprehensive annotation for individual TCDs [34]. The TCM-SAS web application includes a natural language processing (NLP) program that automatically identifies TCD terms in the abstract text of peer-reviewed journal articles which describes TCD-specific knowledge. The identified TCDs in the article abstracts are automatically mapped to our TCDO terms. The TCDO can then provide detailed semantic annotation for the TCD. Meanwhile, the TCDO-based NLP program provides new information to support further annotation for the TCD. A knowledge database is also generated to store the detailed textual and semantic annotation information for each TCD. Such new information can then later be added to the TCDO to improve the semantic annotation of the TCD. The interactive TCM-SAS web page has two roles. First, it supports the standardized annotation of TCDs by automatic NLP searching and annotation. Second, it supports efficient web queries of TCD information.

**Figure 6:**
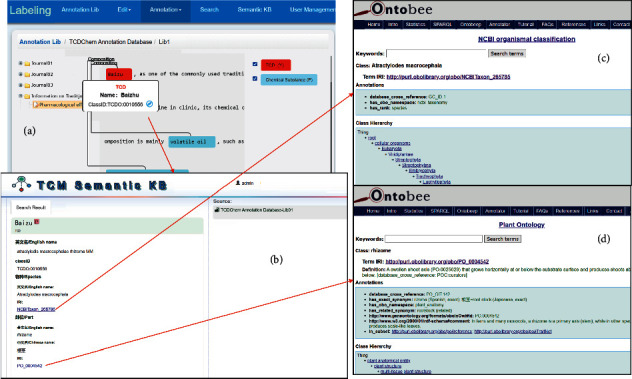
TCDO-supported automatic TCD semantic annotation in TCM-SAS.

As an example of the TCM-SAS system, Figure 6 demonstrates how TCM-SAS annotates Baizhu (i.e., *Atractylodis macrocephalae Rhizoma*, or its Chinese name “白术(药材)”), a medicinal material (MM) prepared from the dry root of the plant *Atractylodes macrocephalus* Koidz. This demonstration shows that TCM-SAS is able to automatically annotate a TCD medicine material (MM) such as Baizhu from peer-reviewed articles such as [35] and identify the MM name and its chemical ingredients from the article. As shown in Figure 6(a), a TCM-SAS page shows the semantic annotation for Baizhu (*Atractylodis macrocephalae Rhizoma* MM) or “白术(药材)” in Chinese. From the abstract of a peer-reviewed article, TCM-SAS automatically identified Baizhu (in red) and its chemical ingredients (in light blue). A click on the Baizhu popped up a screen that explains the TCD with its TCDO ID (TCDO:0010556). Another click on the circular symbol next to the ID led to its corresponding page on the TCM Semantic Knowledge Base (TCM-SKB). In Figure 6(b), this TCM-SKB page provides the structured annotated information for Baizhu, including its Chinese name, English name, plant taxonomy name, and anatomical part. The semantic annotation of the TCD including the taxonomical species of the plant and the part of the plant used for the MM can also be automatically extracted through our linkages to the corresponding pages in the Ontobee ontology linked server [13] (Figures 6(b) and 6(d)). Such information provides consistent and machine-interpretable information for the TCD.

## 4. Discussion

TCDs have been traditionally used to improve and treat various diseases, especially complex diseases such as autoimmune disorders, cardiovascular diseases, infectious diseases, and cancers. TCDs are also a great resource for modern new drug development. However, the information of TCDs is not well organized, inhibiting their effective knowledge representation and analysis. To address this issue, we have developed the TCDO using the state-of-the-art ontology development methods. TCDO represents an original format and representation of the TCD system. TCDO is aimed to become a domain reference ontology for semantically representing basic knowledge of TCDs and supporting various applications. In this manuscript, we demonstrate that TCDO provides the standardized ontological representation of TCD entities, their attributes, plant species, and the logical relations among them, supporting advanced TCD analyses.

TCDO is the first systematical ontological representation of the TCDs using the BFO as the top-level ontology and applying the Open Biological and Biomedical Ontology (OBO) Foundry principles [19]. As introduced in the Introduction section, ontology has emerged to be important to standard data and knowledge representation, sharing, and advanced computer-interpretable AI reasoning and analysis. However, there have not been a well-developed and widely accepted ontology in the first of TCM and TCDs. TCM is a very broad domain that covers traditional Chinese drugs and many other topics such as acupuncture, cupping therapy, bonesetter, and qigong exercise. In this study, we focused on the TCDs so that we can be specific and define this domain more efficiently. TCDs are indeed a major topic of TCM. There have been many properties in TCDs such as nature, flavor, toxicity, and channel tropism. By using the BFO as the top-level ontology framework, we were able to represent them in a rational and meaningful format. While our previous ontological study on traditional Chinese decoction pieces drugs for treating rheumatism is also based on the BFO framework [20], the previous study did not touch base on the root level definitions of these TCD-specific property definitions. In addition, TCDO also provides a much more comprehensive representation of hundreds of traditional Chinese decoction pieces. Using the TCDO platform, we will be able to represent more TCDs in the future.

Another comparison is the ISO standard (ISO/TC 249) of traditional Chinese medicine (https://www.iso.org/committee/598435.html). The TCM ISO covers both traditional and modern aspects of TCM. The quality and safety of the raw materials, manufactured products, and medical devices are currently focused on. Ontology provides a logical and machine-readable format for standard representation. It is possible to use ontology to present various types of TCM aspects. By aligning with BFO, our TCDO aims to eventually cover the TCM drug aspects. We will also later target representing the other aspects represented in the TCM ISO standard such as the quality, safety, and standard manufacturing and usage.

The ontology for Traditional Chinese Medicine Language System (TCMLS), developed by Long et al. [17], aims to integrate existing TCM terminologies through mapping the semantic types to the GFO, a top-level ontology [36]. As a middle-level ontology, TCMLS does not include any specific traditional Chinese drugs. Instead, TCMLS focuses on providing many new relations and semantic approaches in order to support TCD representation. In comparison, TCDO differs from TCMLS in many ways. First, TCDO uses the Basic Formal Ontology (BFO) as the top-level ontology. By aligning with BFO, TCDO is able to quickly align with over 300 other ontologies that also use BFO. Second, unlike TCDO, TCMLS does not include any specific traditional Chinese drugs. However, TCDO has a major focus on representing specific TCDs and their individual properties. Third, in terms of semantic modeling, TCMLS proposes many new relations and semantic representation approaches. However, TCDO primarily uses the semantic relations defined in the OBO Relation Ontology (RO) [37]. Examples of such relations include “part of” and “located in,” which are RO relations commonly and widely used by hundreds of other ontologies. Instead of reinventing the wheel, our direction reuse of these RO relation terms makes TCDO more interoperable. Only when there are no existing relations defined in the reference ontologies, TCDO develops its own relations, which are typically TCD-specific relations (e.g., “derives from medicinal material” TCDO:0000069).

TCDO provides an open and public reference ontology for many application scenarios such as TCD terminology standardization, network pharmacology analysis, and clinical and medical applications. TCDO has been used in different applications. First, TCDO can serve as a knowledge base of TCDs and allow automated reasoning and queries. Based on the formal and logic representation of the knowledge of TCDs, their related entities such as the source plant species, chemical entities, TCD features, and the computer-interpretable logic relations among these entities, we can develop computational queries to automatically query various information from the TCDO knowledge. Second, we can use the internal knowledge logically defined in the TCDO to perform systematic analysis to identify scientific insights. We have applied TCDO to systematically analyze the TCD toxicity and its relation with taxonomy, nature, flavor, and channel tropism, resulting in many insightful results. In addition, TCDO was also used to analyze the correlations between flavor and nature. Such statistical results would be difficult to obtain without the internal logic and hierarchical design of the TCD knowledge representation in TCDO. Furthermore, we have added a new biomedical use case of TCDO, that is, applying TCDO to support the development of a web-based TCD semantic annotation system, which provides comprehensive annotation for individual TCDs so that clinical doctors and biomedical researchers can quickly search and/or annotate individual TCDs. Meanwhile, we also expect that its public availability and future enhancement will support more applications by us and others, and we also look forward to wide collaborations for new program and tool development for different purposes.

With the rapid progress of medical informatics, growing studies have attempted to identify TCD's bioactive ingredients and to clarify their mechanisms of action using integrated data sets from different domains. TCDO ontology provides a format to represent the ingredients of TCDs and link the ingredients to the source organisms (usually plants and sometimes animals) and specific chemical entities. The reuse of existing ontologies, including NCBITaxon and ChEBI, to represent these aspects is the fast and reproducible way to do. Overall, the state-of-the-art ontology development technology [23] is used in this study.

Using the TCDO as a logically represented TCD knowledge base, we have identified several important scientific insights from our use case studies. For example, the study of TCDO toxicity is critical to improve the safety and efficacy of TCD usage. Our Use Case 2 systematically analyzed the relations between TCD toxicity and several TCD properties including their associated plant taxonomy, nature, flavor, and channel tropism. Traditionally, TCD plant taxonomy is based on the Engler system [38]. However, our TCDO uses the NCBITaxon taxonomy ontology for the representation of the source of Chinese medicinal materials. The NCBITaxon taxonomy ontology is derived from the NCBI taxonomy system [39], which uses the modern APG system (Angiosperm Phylogeny Group system) for plant classification, which was released in 2016 [40]. The APG system is different from the Engler and Prantl system used in traditional Chinese medicine in China. The usage of the APG system allows us to integrate our traditional Chinese medicine classification with the newest system, supporting more robust classification and usage. Given the logical and semantic knowledge representation capability, TCDO provides an ideal platform for us to specifically use the APG-based NCBITaxon taxonomy system for the computer-assisted integration and reasoning for the plant classification. To our knowledge, our study is the first systematical analysis of the relations between TCD toxicity and the latest APG-based plant classification.

Using the APG-based taxonomy and our TCDO representation, we found that TCDs with toxic quality have been focused on Ranunculaceae (row 3 in Table 3). Considering that toxic TCDs turn to be effective against some chronic diseases such as cancer [41] and rheumatoid arthritis [42], our statistical results may be useful to predict new TCD toxicity and new effective TCDs for chronic diseases. More investigation is deserved to further identify the differences and similarities of TCD toxicity using the two different taxonomy classification methods.

In addition to the taxonomy study, we also studied the relations between the toxicity and three TCD properties (i.e., nature, flavor, and channel tropism) (Table 1), and we singled out those TCDs with hot TCDs and compared different properties (Table 3). Several new findings were identified. Fu et al. [43] reported their most updated correlation analysis of adverse drug reactions and drug properties in 2019. Many of our findings align with their work, for example, in terms of TCD nature, the high associations between hot TCD and high toxicity and warm/cold TCDs and medium toxicity, and, in terms of TCD flavor, pungent TCDs and medium toxicity and bitter TCDs and low/medium toxicity. Our study found that all 12 tasteless TCDs have no toxicity, which was not reported in their analysis. In terms of channel tropism, both their study and our study found that medium/high toxicity is closely associated with kidney and liver channel [43]. However, our study also found that many TCDs with spleen channel also have high toxicity, and we also found more medium toxicity TCDs being lung channel.

Use Case 3 studies the correlations between ‘TCD nature” (“中药药性” in Chinese, TCDO_0000063) and “TCD flavor” (“中药药味” in Chinese, TCDO_0000064). Each TCD has its own flavor and nature, and many TCD flavors and natures are often closely associated. Consistent with the findings reported by Ye et al. [44], our results also identified three most common flavor-nature associations: pungent-warm, sweet-neutral, and bitter-cold. Based on the TCM theory, pungent and warm TCDs support the expelling of pathogenic factors from the muscles and skin, sweet and neutral TCDs help the supplementing, and bitter and cold TCDs support heat-clearing [45]. Meanwhile, our study made several new findings not reported in [44], including the warm-bitter and sweet-cold associations. Based on the TCM theory, the bitter and warm-natured drugs (e.g., *Atractylodes Rhizome*苍术、officinal magnolia bark厚朴 and dried tangerine peel陈皮) support the dispelling of dampness [46]. In the theory of warm disease, the sweet and cold-natured TCDs (e.g., reed rhizome芦根, henon bamboo leaf 竹叶, chrysanthemum flower菊花) can be applied to clear heat and moisturize the body [45]. Therefore, our findings correlate with the classical TCD theories and meanwhile provide particular TCD examples for further deep investigation on the underlying mechanisms.

Furthermore, relying on the logics and semantics presented in the TCDO OWL ontology, new programs and tools can be developed. Our Use Case 4 demonstrates how TCDO can be used to support a semantic annotation system for TCM Literature [34] as a standard terminology and highlight the usage of the TCDO for semantic search of annotation results of TCM literature. We envision that the future applications of TCDO may include the standardization and mining of electronic health records, pharmacological analysis, and broadly integration of information from other domains, such as pharmacy, molecular biology, and biochemistry. We also welcome collaborations from the community to further develop and apply the TCDO.

## 5. Conclusions

To support systematical and computer-interpretable knowledge representation and integration of traditional Chinese drugs (TCDs), we have developed and evaluated the applications of the community-based TCD ontology (TCDO). The TCDO is developed by aligning with the Basic Formal Ontology (BFO), an ISO-approved top-level ontology. Important TCD-related terms including TCD, high-level TCD categories, and the properties of TCDs (i.e., nature, flavor, toxicity, and channel tropism) are defined with textual and logical definitions. TCDO also systematically represents more than 400 popular TCD-DPs and CMMs. Specific Description Logic (DL) and SPARQL queries are demonstrated for efficient computer-assisted TCDO knowledge query. Our statistical analysis of the TCDO knowledge revealed scientific insights in terms of TCD medium or high toxicity in different taxonomical hierarchies of plants. The associations between TCD toxicity and other features (including nature, flavors, and channel tropism) were systematically and statistically analyzed. Three pairs of TCD flavor-nature associations (i.e., bitter-cold, pungent-warm, and sweet-neutral) were identified. Furthermore, TCDO has been used to support the development of a web-based traditional Chinese medicine semantic annotation system for comprehensive annotation for individual TCDs. As a new interoperable ontology in the domain of traditional Chinese drugs, TCDO will be further developed in the future to support more advanced AI applications and facilitate the improvement of public health.

## Figures and Tables

**Table 1:** Relations between TCD toxicity and properties.

Property category	Subcategory	Count				Jaccard index		
		Low toxicity	Medium toxicity	High toxicity	All	Low toxicity	Medium toxicity	High toxicity
Nature	Cold (TCD)	7	14	1	160	0.042	0.077^*∗*^	0.006
	Cool (TCD)	1	1	0	29	0.023	0.016	0.000
	Neutral (TCD)	3	5	0	92	0.029	0.041	0.000
	Warm (TCD)	5	15	2	145	0.032	0.090	0.013
	Hot (TCD)	1	2	5	15	0.034	0.041	0.278

Flavor	Astringent (TCD)	0	3	0	38	0.000	0.042	0.000
	Bitter (TCD)	12	16	4	202	0.059	0.072	0.019
	Pungent (TCD)	7	21	7	168	0.040	0.115	0.041
	Salty (TCD)	2	1	0	47	0.033	0.012	0.000
	Sour (TCD)	0	3	0	31	0.000	0.047	0.000
	Sweet (TCD)	0	5	0	184	0.000	0.023	0.000
	Tasteless (TCD)	0	0	0	12	0.000	0.000	0.000

Channel tropism	Bladder channel	1	0	0	36	0.020	0.000	0.000
	Gallbladder channel	0	1	0	22	0.000	0.018	0.000
	Heart channel	0	7	1	104	0.000	0.053	0.009
	Kidney channel	3	11	4	122	0.022	0.075	0.032
	Large intestine channel	2	8	1	74	0.023	0.078	0.012
	Small intestine channel	1	1	0	21	0.029	0.018	0.000
	Liver channel	11	18	6	221	0.049	0.075	0.027
	Lung channel	1	17	2	168	0.005	0.091	0.011
	Pericardium channel	0	0	0	6	0.000	0.000	0.000
	Spleen channel	4	11	4	121	0.030	0.075	0.032
	Stomach channel	4	8	2	147	0.025	0.046	0.013
	Triple energizer channel	0	0	0	3	0.000	0.000	0.000

Total		15	36	8				

*Note.*^*∗*^Red color is for a Jaccard index value >0.1 and orange for a Jaccard index value of 0.05–0.1.

**Table 2:** Plant taxonomic distribution of representative TCDs and their toxicities.

Taxonomic hierarchy						No toxicity	Low toxicity	Medium toxicity	High toxicity
Eukaryota						351	14	30	6
	Acrogymnospermae					8	0	2	0
	Mesangiospermae					300	10	24	5
		Eudicotyledons				223	9	17	5
			Ranunculales			14	0	0	0
			Papaveroideae			0	0	1	0
			*Aconitum*			0	0	1	1
			Pentapetalae			210	9	15	4
				Asterids		95	4	5	1
					Campanulids	47	3	1	0
					Cornales	2	0	1	0
					Ericales	3	0	0	0
					Lamiids	44	1	3	1
				Caryophyllales		19	0	1	0
				Rosids		87	5	9	3
					Malvids	29	3	2	0
					Fabids	57	2	7	3
					*Ampelopsis japonica*	1	0	0	0
				Santalales		2	0	0	0
				Saxifragales		8	0	0	0
		Liliopsida				61	1	6	0

*Note.* The numbers represent the number of TCDs that are categorized as no toxicity, low toxicity, medium toxicity, and high toxicity.

**Table 3:** 15 hot TCDs and their properties.

TCD drug	Chinese label	Taxonomy	Flavor	Channel tropism	Toxicity
Arsenic sublimate DP	砒石(饮片)	Mineral	Pungent	Liver, lung	High
Common monkshood mother root DP	川乌(饮片)	*Aconitum*	Pungent, bitter	Heart, kidney, liver, spleen	High
Defatted croton seed powder DP	巴豆霜(饮片)	Pentapetalae	Pungent	Large intestine, stomach	High
*Mylabris* DP	斑蝥(饮片)	Holometabola	Pungent	Kidney, liver, stomach	High
Red oxide of mercury DP	红粉(饮片)	Mineral	Pungent	Lung, spleen	High
Camphor DP	樟脑(饮片)	Magnoliidae	Pungent	Heart, spleen	Medium
Common *Curculigo* rhizome DP	仙茅(饮片)	Pentapetalae	Pungent	Kidney, liver, spleen	Medium
Medicinal *Evodia* fruit DP	吴茱萸(饮片)	Pentapetalae	Pungent, bitter	Kidney, liver, spleen, stomach	Low
Alpiniae officinarum rhizoma DP	高良姜(饮片)	Petrosaviidae	Pungent	Spleen, stomach	None
Cassia bark DP	肉桂(饮片)	Magnoliidae	Pungent, sweet	Heart, kidney, liver, spleen	None
Dried ginger DP	干姜(饮片)	Zingiberaceae	Pungent	Heart, kidney, lung, spleen, stomach	None
Piperis fructus DP	胡椒(饮片)	Magnoliidae	Pungent	Large intestine, stomach	None
Piperis longi fructus DP	荜茇(饮片)	Magnoliidae	Pungent	Large intestine, stomach	None
Prepared dried ginger DP	炮姜(饮片)	Zingiberaceae	Pungent	Liver, spleen, stomach	None
Testiset penis phocae DP	海狗肾(饮片)	Laurasiatheria	Salty	Kidney	None

**Table 4:** Relations between TCD natures and flavors.

	Count						Jaccard index				
	Cold (TCD)	Cool (TCD)	Neutral (TCD)	Warm (TCD)	Hot (TCD)	Total	Cold (TCD)	Cool (TCD)	Neutral (TCD)	Warm (TCD)	Hot (TCD)
Astringent (TCD)	10	6	15	6	0	37	0.053	0.100	0.132	0.034	0.000
Bitter (TCD)	98	15	30	57	2	202	0.371^*∗*^	0.069	0.114	0.197	0.009
Pungent (TCD)	30	7	22	95	14	168	0.101	0.037	0.092	0.436	0.083
Salty (TCD)	24	1	10	11	1	47	0.131	0.013	0.078	0.061	0.016
Sour (TCD)	10	0	10	11	0	31	0.055	0.000	0.088	0.067	0.000
Sweet (TCD)	56	22	57	48	1	184	0.194	0.115	0.260	0.171	0.005
Tasteless (TCD)	7	2	3	0	0	12	0.042	0.051	0.030	0.000	0.000
Total	160	29	92	145	15						

*Note.*^*∗*^Jaccard index with its value > 0.2 is highlighted in red color.

## Data Availability

TCDO ontology source is freely available at GitHub (https://github.com/TCMOntology/TCDO).

## Abbreviations:

AE: Adverse event
BFO: Basic Formal Ontology
ChEBI: Chemical Entities of Biological Interest
DL: Description Logic
DrON: Drug ontology
NCBI: National Center for Biotechnology Information
NCBITaxon: NCBI taxonomy ontology
OAE: Ontology of Adverse Events
OCMR: Ontology of Chinese Medicine against Rheumatism
OBO: Open Biological and Biomedical Ontology
OGMS: Ontology for General Medical Science
OWL2: Web Ontology Language
PO: Plant ontology
RDF: Resource description framework
RO: Relation Ontology
TCD: Traditional Chinese drug
TCDO: Traditional Chinese drug ontology
TCM: Traditional Chinese medicine.

## Supplementary Materials

Supplementary Materials Supplemental Table 1: information of 8 key terms of TCDO: name, name in Chinese, ID, and text definitions.
