# Supplementary material for: TCDO: A Community-Based Ontology for Integrative Representation and Analysis of Traditional Chinese Drugs and Their Properties
Source: Evid Based Complement Alternat Med. 2021 Sep 23;2021:6637810. doi: 10.1155/2021/6637810 (PMC8483929; doi:10.1155/2021/6637810)
Supplement: Supplementary Materials — Supplemental Table 1: information of 8 key terms of TCDO: name, name in Chinese, ID, and text definitions. [file 6637810.f1.pdf]

term name	term name in Chi	term ID	text definition
traditional Chinese drug (TCD)	传统中药	TCDO_0000001	A drug that is developed originally from ancient China. A TCD is a drug derived from the usage of medicinal material grown and
decocting pieces	中药饮片	TCDO_0000002	A traditional Chinese drug that is prepared with crude medicine as raw materials. According to Chinese medicine theory, after processing, it can be directly used in traditional Chinese medicine clinical or pharmaceutical production and use of prescription drugs. It
Chinese patent drug	中成药	TCDO_1000000	A traditional Chinese drug that is modernized into a ready-to-use form, such as tablets, oral solutions, or dry suspensions.
medicinal material	中药药材	TCDO_1000068	A material entity that is rude natural medicinal for processing and preparing traditional Chinese drugs. Medicinal materials are medicinal parts of medicinal plants, animals and minerals after preliminary processing, which are used as raw materials to make decoction pieces in Chinese medicines. It is also called Chinese crude
TCD nature	中药药性	TCDO_0000063	A function of TCD that induces cold and heat changes in the body according to the cold or heat property of the diseases treated based on
TCD flavor	中药药味	TCDO_0000064	A flavor that reflects the common function of decocting pieces in highly concentrated and abstracted way.
channel tropism	归经	TCDO_0000062	A disposition that the TCD tends to have therapeutic effects on the pathological changes in one or several certain channels. It is also
TCD toxicity	中药毒性	TCDO_0000065	A quality that represents the level of critical or lethal reaction to a dosage of a TCD drug medication.
